# Efficacy of a Binuclear Cyclopalladated Compound Therapy for Cutaneous Leishmaniasis in the Murine Model of Infection with Leishmania amazonensis and Its Inhibitory Effect on Topoisomerase 1B

**DOI:** 10.1128/AAC.00688-17

**Published:** 2017-07-25

**Authors:** Angela Maria Arenas Velásquez, Willian Campos Ribeiro, Vutey Venn, Silvia Castelli, Mariana Santoro de Camargo, Renata Pires de Assis, Rodrigo Alves de Souza, Aline Rimoldi Ribeiro, Thaís Gaban Passalacqua, João Aristeu da Rosa, Amanda Martins Baviera, Antonio Eduardo Mauro, Alessandro Desideri, Elmo Eduardo Almeida-Amaral, Marcia A. S. Graminha

**Affiliations:** a São Paulo State University (UNESP), School of Pharmaceutical Sciences, Araraquara, Brazil; b São Paulo State University (UNESP), Institute of Chemistry, Araraquara, Brazil; c University of Rome, Tor Vergata, Rome, Italy; d Campinas State University (UNICAMP), Biology Institute, Campinas, Brazil; e Instituto Oswaldo Cruz, Fundação Oswaldo Cruz (FIOCRUZ), Rio de Janeiro, Brazil

**Keywords:** cyclopalladated complex, leishmaniasis, Chagas disease, Leishmania amazonensis, Trypanosoma cruzi, Leishmania donovani, topoisomerase 1B

## Abstract

Leishmaniasis is a disease found throughout the (sub)tropical parts of the world caused by protozoan parasites of the Leishmania genus. Despite the numerous problems associated with existing treatments, pharmaceutical companies continue to neglect the development of better ones. The high toxicity of current drugs combined with emerging resistance makes the discovery of new therapeutic alternatives urgent. We report here the evaluation of a binuclear cyclopalladated complex containing Pd(II) and *N*,*N*′-dimethylbenzylamine (Hdmba) against Leishmania amazonensis. The compound [Pd(dmba)(μ-N_3_)]_2_ (CP2) inhibits promastigote growth (50% inhibitory concentration [IC_50_] = 13.2 ± 0.7 μM) and decreases the proliferation of intracellular amastigotes in *in vitro* incubated macrophages (IC_50_ = 10.2 ± 2.2 μM) without a cytotoxic effect when tested against peritoneal macrophages (50% cytotoxic concentration = 506.0 ± 10.7 μM). In addition, CP2 was also active against T. cruzi intracellular amastigotes (IC_50_ = 2.3 ± 0.5 μM, selective index = 225), an indication of its potential for use in Chagas disease therapy. *In vivo* assays using L. amazonensis-infected BALB/c showed an 80% reduction in parasite load compared to infected and nontreated animals. Also, compared to amphotericin B treatment, CP2 did not show any side effects, which was corroborated by the analysis of plasma levels of different hepatic and renal biomarkers. Furthermore, CP2 was able to inhibit Leishmania donovani topoisomerase 1B (*Ld*topo1B), a potentially important target in this parasite. (This study has been registered at ClinicalTrials.gov under identifier NCT02169141.)

## INTRODUCTION

Neglected tropical diseases affect more than one billion people worldwide (http://www.who.int/neglected_diseases/diseases/en/). According to the World Health Organization, the disorders caused by parasites of Leishmania spp. and T. cruzi (family Trypanosomatidae, order Kinetoplastida) that cause leishmaniasis and Chagas disease, respectively, are both considered neglected diseases since they are more prevalent among impoverished areas and are often overlooked by drug developers and other important players involved in drug access, such as officials in governments and public health programs.

Leishmaniasis is an insect-borne disease caused by more than 20 species of Leishmania and can be manifested as cutaneous, mucocutaneous, or visceral forms. These parasitic diseases affect about 12 million people and endanger another 350 million (1). The cutaneous form is the most common manifestation of the disease with 0.7 to 1.3 million new cases annually, 95% of which occur in the Americas. The current treatment for leishmaniasis poses limitations such as toxicity, a difficult route of administration, and a lack of efficacy in areas where leishmaniasis is endemic due to the emergence of drug resistance and ineffective parasite and vector control (2). All currently available drugs against Leishmania were initially developed for other diseases and have limitations when used to treat leishmaniasis. Despite efforts to discover new drugs against Leishmania spp., either through synthesis or from natural sources (3, 4), treatments are still based on the use of pentavalent antimonials, amphotericin B, miltefosine, pentamidine, or paromomycin, none of them is ideal.

The first-line treatment involves the pentavalent antimonials sodium stibogluconate and meglumine antimoniate, which display severe side effects, including nausea, diarrhea, skin rashes, hepatotoxicity, and cardiotoxicity and are associated with high death rates in HIV-coinfected patients. The polyene antibiotic amphotericin B and its liposomal formulation are second-line therapies and are highly effective in antimony-unresponsive patients, but they have limitations due to the compound's renal toxicity. Although liposomal amphotericin B is currently the most effective strategy, this drug is quite expensive, resulting in a high cost-effectiveness ratio (5). Paromomycin and pentamidine treatments have variable efficacy in different countries, but the use and availability of these drugs in regions where leishmaniasis is endemic is limited (2, 6). Miltefosine is the first orally administered treatment made available for leishmaniasis (7–9). Although it displays high efficacy in adults and children, there are many issues around its use, including potential teratogenicity, prolonged periods of treatment, severe side effects, and drug resistance (2, 10, 11).

The antiprotozoan activity of many metal-based compounds has been investigated and may offer new therapeutic options (12–18). Cyclopalladated compounds have been postulated as potential alternatives for leishmaniasis treatment (14–16). Some mechanistic studies described in the literature have shown that different metal compounds can inhibit the activity of DNA topoisomerase type 1 or 2 (19–22). DNA topoisomerases are ubiquitous enzymes that play important roles in cells such as DNA replication, transcription, recombination, and repair and have been identified in trypanosomatids (23–25). The DNA topoisomerase is essential for parasite viability, and the enzyme has been reported as a promising target for parasitic diseases (23–25). Type I DNA topoisomerase (Topo1) have been isolated from Leishmania donovani (26), T. cruzi (27), and Trypanosoma brucei (28), and the L. donovani and T. cruzi enzymes have been structurally characterized (25–27, 29). Among these protozoa, topoisomerase Topo1B is highly similar (89 to 95% amino acid sequence identity), but the parasite enzyme is structurally and/or functionally different from its human counterpart, making it suitable for drug design (25). Indeed, some DNA topoisomerases inhibitors include the anti-leishmanial compounds sodium stibogluconate and urea stibamine (23, 30).

In this work, we evaluated the antileishmanial *in vitro* and *in vivo* efficacy of the binuclear cyclopalladated complex CP2 and its potential spectrum of action against T. cruzi, as well as its possible mechanism of action involving DNA topoisomerase 1B inhibition.

## RESULTS

### Determination of the IC_50_ toward the insect stages of parasites.

**FIG 1:**
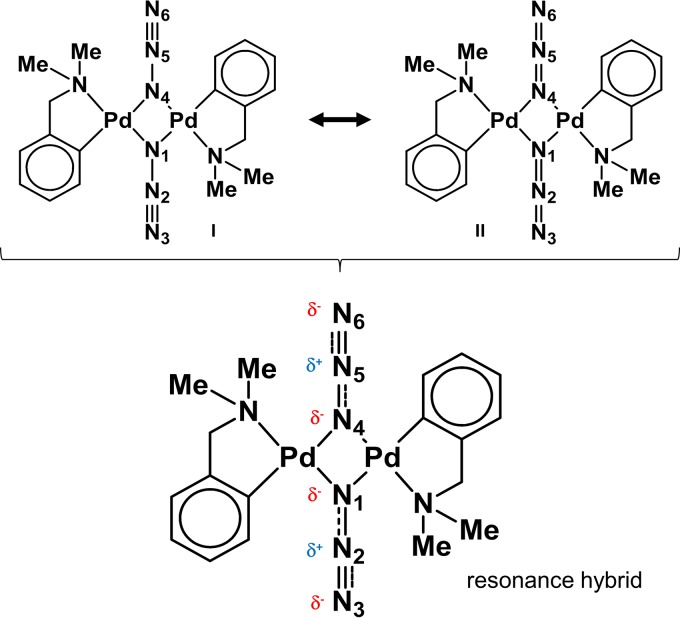
Structure of binuclear cyclopalladated complex CP2, [Pd(dmba)(μ-N_3_)]_2_ (61). A better model for [Pd(dmba)(N_3_)]_2_ is a blend of Lewis structures I and II with N1-N2 and N4-N5 intermediate in properties between a single and a double bond, with N2-N3 and N5-N6 between a double and a triple bond. This blending of structures is called resonance. The blended structure is a resonance hybrid of the contributing Lewis structures. A molecule does not flicker between different structures: a resonance hybrid is a blend of structures.

The antiprotozoal activity of CP2 (Fig. 1) was initially determined against the insect stages of L. amazonensis and T. cruzi, which are actively replicating in culture. It was determined that CP2 was biologically active against the promastigote and epimastigote forms of L. amazonensis and T. cruzi, respectively. The 50% inhibitory concentration (IC_50_) for L. amazonensis promastigotes (13.2 μM) and T. cruzi epimastigotes (7 μM) showed that CP2 is approximately 2-fold more active than amphotericin B (23.1 μM) and has similar activity as benznidazole (4.1 μM), as shown in Table 1.

**TABLE 1:** Antiparasitic activities, mammalian cell toxicities, and selective indices of CP2^*a*^

Compound	CC_50_ (μM) ± SD for macrophages	Avg IC_50_ (μM) ± SD^*b*^			
		L. amazonensis		T. cruzi	
		Promastigote	Amastigote	Epimastigote	Amastigote
CP2	506.0 ± 10.7	13.2 ± 0.7 (38.5)	10.1 ± 2.2 (50)	7.0 ± 0.9 (73.5)	2.3 ± 0.5 (225)
Pentamidine	35.7 ± 6.9	7.6 ± 0.1 (4.78)	5.1 ± 1.1 (7.1)		
Benznidazole	988.4 ± 38.1			4.1 ± 0.3 (243)	5.3 ± 1.4 (187.2)
Amphotericin B	23.1 ± 2.5	3.2 ± 0.1 (7.2)	4.9 ± 0.1 (4.7)		

a Antiparasitic activities are expressed as half-maximal inhibitory concentrations (IC_50_), and mammalian cell toxicities are expressed as half-maximal cytotoxic concentrations (CC_50_).

b The selective index (indicated in parentheses) was calculated as the CC_50_/IC_50_ of CP2. *P* < 0.05 for all values.

### Effect of CP2 on murine macrophages and mammary adenocarcinoma cells LM3.

The cytotoxicity of CP2 was evaluated against murine macrophages or LM3 cells. The results showed that CP2 presented lower cytotoxicity against macrophages (CC_50_ = 506.0 ± 10.7 μM), as well as LM3 cells (CC_50_ = 71.0 ± 4.0 μM), compared to the reference drugs pentamidine (CC_50_ = 35.7 ± 6.8 μM), amphotericin B (23.1 ± 2.5 μM), benznidazole (988.4 ± 38.1 μM), or cisplatin (30.3 ± 3.7 μM).

### Effect of CP2 on L. amazonensis and T. cruzi intracellular amastigote forms.

We further evaluated whether CP2 presented antiprotozoal activity against the intracellular amastigotes of L. amazonensis and T. cruzi, the clinically most relevant life cycle stages of the parasites in leishmaniasis and Chagas disease, respectively. Although the potency of CP2 (IC_50_ = 10.1 ± 2.2 μM; selective index [SI] = 49.9) for L. amazonensis intracellular amastigotes was two times lower than those of pentamidine (IC_50_ = 5.1 ± 1.1 μM; SI = 7.1) and amphotericin B (IC_50_ = 4.9 ± 0.1 μM; SI = 4.7), its selectivity to the parasite (SI = 50) was at least 10 times higher than that of the positive-control drugs. The cyclopalladated CP2 (IC_50_ = 2.3 ± 0.5 μM; SI = 224.9) was two times more potent than benznidazole (IC_50_ = 5.3 ± 1.4 μM; SI = 187.2) and highly selective to the parasite (Table 1).

**FIG 2:**
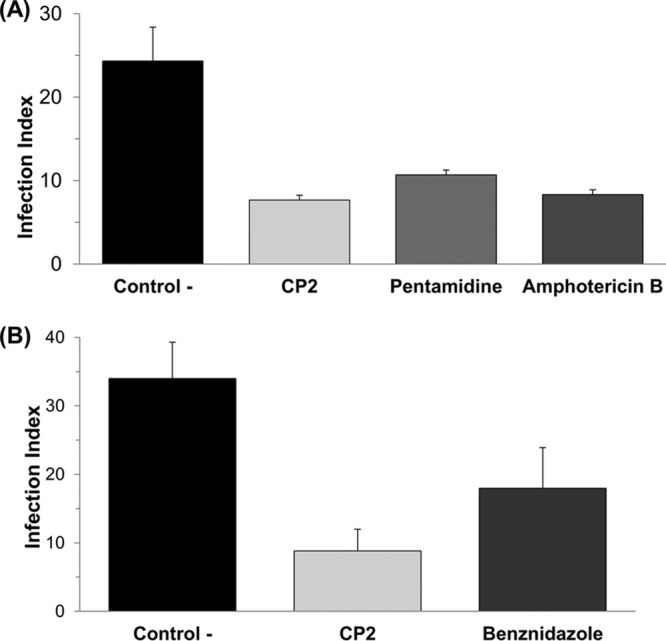
*In vitro* effect of CP2, pentamidine, and amphotericin B on L. amazonensis intracellular amastigotes (A) and *in vitro* effect of CP2 and benznidazole on T. cruzi intracellular amastigotes (B). The infection index was calculated after 72 h of treatment with the IC_50_s of each compounds. The negative control is L. amazonensis or T. cruzi intracellular amastigotes not treated with CP2. Data are expressed as averages plus the standard deviations (SD) for three independent experiments (*P* < 0.05).

Regarding the infection index (the percentage of infected cells × the number of the intracellular parasites), treatment with CP2 caused 68.5 and 74% reductions in the number of intracellular amastigotes parasites for L. amazonensis and T. cruzi, respectively (Fig. 2), while pentamidine and amphotericin B for L. amazonensis and benznidazole for T. cruzi caused 56, 65, and 47% parasite reductions, respectively.

### Effect of CP2 on BALB/c mice infected with L. amazonensis.

**FIG 3:**
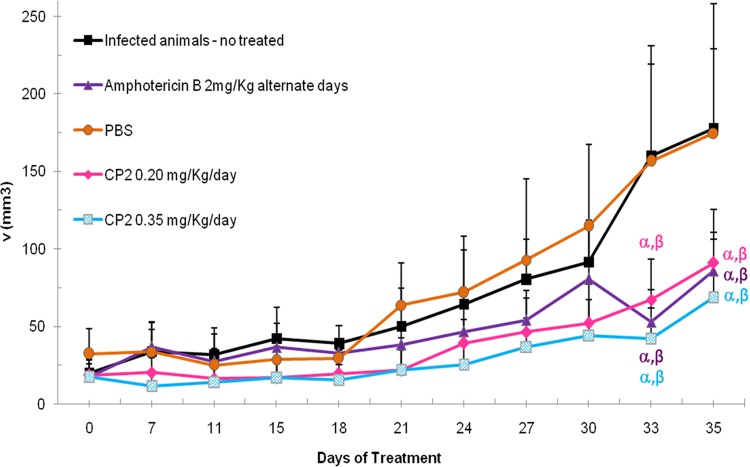
*In vivo* efficacies of CP2 and amphotericin B treatment in BALB/c mice infected with L. amazonensis. Development of foot lesions in L. amazonensis-infected BALB/c mice treated with CP2. The treatment was started 60 days after infection and continued for 35 days. Data points represent the average measurements for seven groups of eight mice each. The development of foot lesions was monitored three times a week. Values indicate the mean volume (volume *= D* × *d* × *e*, where *D* is the larger diameter, *d* is the minor diameter, and *e* is the thickness) of lesions in each group, and bars represent the SD. α, statistically significant difference from infected, nontreated animals (*P* < 0.05); β, statistically significant difference from infected animals treated with PBS (*P* < 0.05).

BALB/c mice infected with L. amazonensis (8 weeks postinfection) were treated for 35 days on a daily basis with two different doses of CP2 (0.2 or 0.35 mg/kg/day). A group of infected animals were treated with amphotericin B (2 mg/kg/day) on a daily basis from days 1 to 10, followed by an alternate base treatment from days 11 to 35. After 33 days of treatment, the animals treated with CP2 at both tested doses or with amphotericin B presented decreased lesion sizes compared to infected controls (animals infected and untreated and animals infected and treated with phosphate-buffered saline [PBS]; *P* < 0.05) as indicated in Fig. 3.

**FIG 4:**
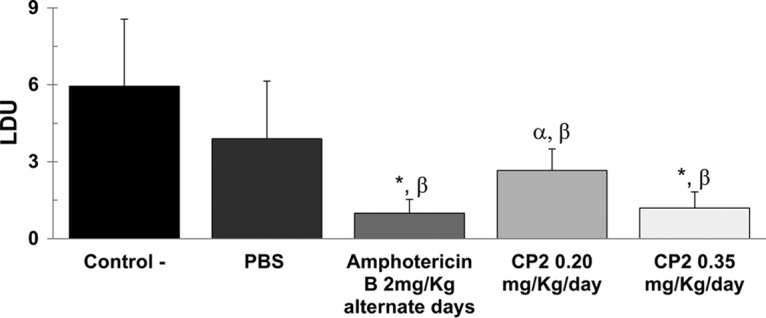
Quantitation of tissue parasite load of L. amazonensis in skin lesions of infected BALB/c mice using the LDU index (i.e., the number of Leishmania amastigotes in 1,000 nucleated cells per organ weight). The data are expressed as averages plus the SD. *, α, statistically significant difference relative to the control group (*P* ≤ 0.001, *P* ≤ 0.01); β, statistically significant difference relative to the group treated with PBS (*P* < 0.05). The negative controls were infected animals not treated with CP2.

Regarding the parasite burden, BALB/c mice treated with 0.2 or 0.35 mg/kg/day of CP2 showed less skin parasitism in a dose-dependent manner compared to infected controls (*P* < 0.05). This represents decreases in parasitemia of 55 or 80% as determined from the Leishman-Donovan units (LDU index), whereas treatment with amphotericin B at 2 mg/kg/day of caused a similar reduction (83%) compared to CP2 at 0.35 mg/kg/day (*P* < 0.05) (Fig. 4).

### Biomarkers of hepatic and renal function.

**FIG 5:**
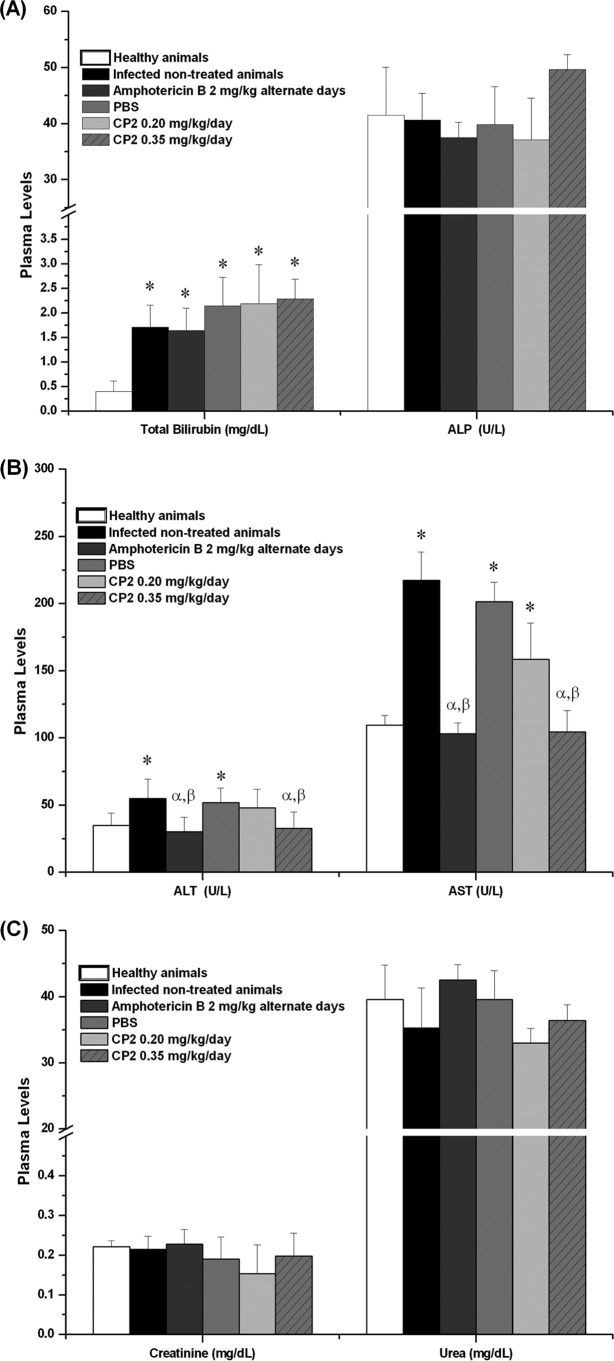
Plasma levels of biomarkers of liver and renal function in BALB/c mice noninfected and infected with L. amazonensis and treated with 0.20 or 0.35 mg/kg/day of CP2. (A) Total bilirubin and ALP levels; (B) ALT and AST levels; (C) urea and creatinine levels. The data are expressed as averages plus the SD. *, statistically significant difference with the noninfected animals (healthy animals) (*P* < 0.05); α, statistically significant difference with infected, untreated animals (*P* < 0.05); β, statistically significant difference with infected animals treated with PBS (*P* < 0.05).

Changes in liver function due to infection or different treatments were monitored by measuring the plasma levels of total bilirubin, alkaline phosphatase (ALP), alanine aminotransferase (ALT), and aspartate aminotransferase (AST). Increased circulating levels of bilirubin were found in plasma of animals infected with L. amazonensis. For infected animals treated with amphotericin B or with CP2, no changes were observed in the bilirubin levels in compared to untreated infected animals (Fig. 5A). No differences were found in the plasma ALP levels among all studied groups (Fig. 5A).

Increased levels of ALT and AST were found in animals infected with L. amazonensis (untreated or treated with PBS). Treatment with amphotericin B prevented the liver damage of infected animals, since the ALT and AST levels were low. For infected animals treated with 0.35 mg/kg/day of CP2, both the ALT and the AST levels were similar to those of uninfected animals and of infected animals treated with amphotericin B (Fig. 5B). The evaluation of the renal function did not show significant changes for creatinine or urea levels in plasma of all animals (Fig. 5C).

### *In silico* prediction of CP2 pharmacokinetic and toxicity properties.

The ADMET (absorption, distribution, metabolism, excretion, and toxicity) properties of CP2 demonstrated that the cyclopalladated presented 92.96% predicted permeability for human intestinal absorption according to the data analyses presented in Table 2.

**TABLE 2:** Predicted ADMET properties of CP2 determined using the admetSAR tool

Predicted ADMET properties of CP2	CP2	
	Result	Probability (%)
Absorption		
Blood-brain barrier	Yes	87.19
Human intestinal absorption	Yes	92.96
P-glycoprotein substrate	Substrate	72.67
P-glycoprotein inhibitor I	Noninhibitor	67.06
P-glycoprotein inhibitor II	Noninhibitor	55.47
Metabolism		
CYP450 2C9 substrate	Nonsubstrate	86.50
CYP450 2D6 substrate	Nonsubstrate	78.46
CYP450 3A4 substrate	Substrate	57.82
CYP450 1A2 inhibitor	Noninhibitor	76.55
CYP450 2C9 inhibitor	Noninhibitor	72.79
CYP450 2D6 inhibitor	Noninhibitor	81.01
CYP450 2C19 inhibitor	Noninhibitor	66.75
CYP450 3A4 inhibitor	Noninhibitor	82.60
Excretion		
Renal OCT2	Noninhibitor	53.74
Toxicity		
hERG inhibitor	Noninhibitor	59.08
Carcinogens	Noncarcinogens	82.09
Acute oral toxicity^*a*^	III	51.82
Rat acute toxicity (mol/kg)	2.7317	

a Acute oral toxicity compounds were classified into four categories based on the criterion of U.S. Environmental Protection Agency (category III includes compounds with LD_50_ values of >500 mg/kg but <5,000 mg/kg).

### Inhibitory effect of CP2 on L. donovani topoisomerase 1B.

**FIG 6:**
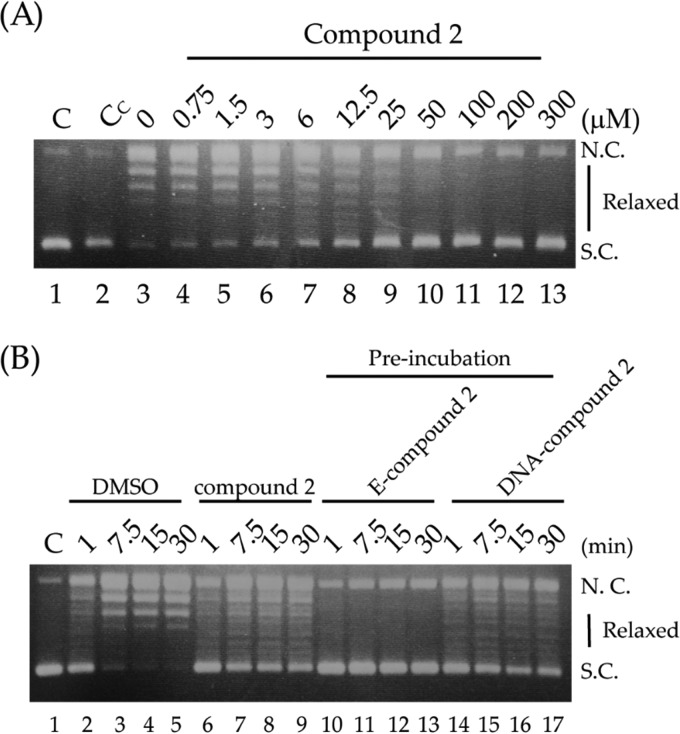
(A) Relaxation of negative supercoiled plasmid DNA by L. donovani topoisomerase 1B (*Ld*topo1B) in the presence of increasing concentrations of CP2 (lanes 4 to 13). Lane 1, only substrate; lane 2, substrate plus 300 μM CP2; lane 3, substrate plus *Ld*topo1B enzyme. (B) Plasmid relaxation as a function of time in DMSO (at a concentration identical to that used to dissolve the CP2 compound) (lanes 2 to 5), 12.5 μM CP2 (lanes 6 to 9), 12.5 μM CP2 preincubated for 5 min with enzyme before DNA addition (lanes 10 to 13), 12.5 μM CP2 preincubated for 5 min with DNA before enzyme addition (lanes 14 to 17). NC, nicked circular plasmid DNA; SC, supercoiled plasmid DNA.

The effect of CP2 on *Ld*topo1B activity was analyzed by a supercoiled plasmid relaxation assay (Fig. 6A). The assay allows determination of the conversion of supercoiled plasmid DNA to relaxed forms due to the enzyme activity (Fig. 6A, compare lanes 1 and 3). In the presence of CP2, *Ld*topo1B was characterized by a reduced relaxation capability of the substrate. The inhibition starts at a 3 μM concentration and becomes almost complete at 100 μM (Fig. 6A). Preincubation of the compound with the enzyme before substrate addition increased the efficiency of CP2, since a concentration of 12.5 μM was sufficient to completely inhibit the Leishmania enzyme (Fig. 6B, lanes 10 to 13). This result indicates that CP2 inhibition occurs through a direct interaction with the enzyme, likely binding at the active site although an indirect inhibition cannot be ruled out. Preincubation of the compound with DNA, before enzyme addition, did not affect the relaxation of the substrate by the enzyme (Fig. 6B, lanes 14 to 17). As a control, we performed the experiments in the presence of dimethyl sulfoxide (DMSO) at the same concentration as when added CP2 to the enzyme solution to demonstrate that, at this concentration of DMSO, the enzyme still works perfectly well (Fig. 6A, lane 3, and Fig. 6B, lanes 2 to 5).

### CP2 inhibits the cleavage step in the enzyme's catalytic cycle.

**FIG 7:**
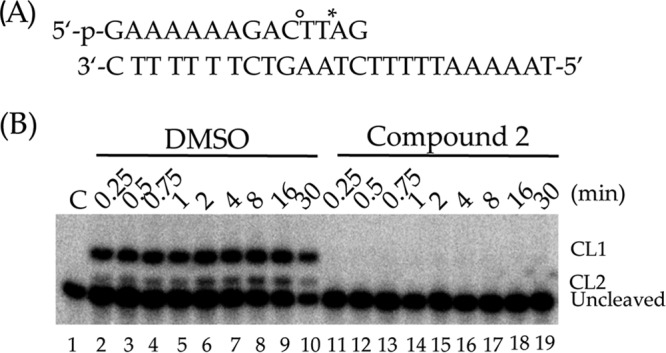
(A) CL1/CL2 suicide substrate used to measure the cleavage kinetics of the enzyme. *, preferential cleavage site (CL1); °, second cleavage site (CL2). (B) Gel analysis of *Ld*topo1B cleavage kinetics in the absence (lanes 2 to 10) or presence (lanes 11 to 19) of 150 μM CP2 at various time points (0, 0.25, 0.5, 0.75, 1, 2, 4, 8, 16, and 30 min). CL1, DNA fragment cleaved at the preferred site; CL2, second cleavage site.

The cleavage rate of *Ld*topo1B was tested in a time course experiment incubating the enzyme with a linearized, partially duplex DNA substrate (Fig. 7A), labeled at its 5′ end with [γ-^32^P]ATP. The substrate is named “suicide substrate” because, after cutting at the preferential cleavage sites (CL1 and CL2), the di/tetranucleotide generated cannot be religated, and the enzyme remains covalently bound to the DNA (cleavage complex). The cleavage reaction is very fast in the absence of CP2; in fact, the bands corresponding to the cleavage complexes are fully formed after 15 s and remain constant as a function of time (Fig. 7B, lanes 2 to 10). In the presence of 150 μM CP2, the cleavage complex bands are not any more visible, indicating that *Ld*topo1B is fully inhibited (Fig. 7B, lanes 11 to 19), permitting us to conclude that CP2 fully inhibits the cleavage reaction of Leishmania topoisomerase.

## DISCUSSION

The discovery of new, safe, and effective antileishmanial therapeutic agents is urgent because most currently used drugs have many problems that make treatment difficult, such as variable efficacy, severe side effects, long-term therapy, and high costs. The discovery of the antikinetoplastid activity of cisplatin (31) and its subsequent clinical development have pointed out that the chemotherapeutic properties of transition-metal complexes and the coordination of different ligands to metal is a possible strategy for finding new antileishmanial drugs. Indeed, several scaffolds of compounds containing palladium (17, 18, 32–34), platinum (35), gold (36), iridium (37), rhodium (38), and iron (39, 40) have shown antikinetoplastid activities.

The use of cyclopalladated complexes is preferable because of their higher thermodynamic and kinetic stability compared to other palladium(II) compounds whose fast hydrolysis and dissociation in solution lead to highly reactive species that are unable to reach the pharmacological targets (34). In this context, several research groups have explored the potential therapeutic applications of cyclopalladated complexes (34, 41–46). Regarding kinetoplastid diseases, it has been shown that cyclopalladated complexes were efficacious in reducing the parasite load in an *in vivo* leishmanial cutaneous model (18) and exhibited trypanocidal potential for Chagas disease treatment (17). More recently, Velásquez et al. showed the leishmanicidal and trypanocidal activities of a series of cyclopalladated compounds of the general formula [Pd(μ-X)(*C*^*2*^,*N*-dmba)]_2_ and [Pd(μ-X)(*C*^*2*^,*N*-dmba)(isn)], (*C*^*2*^,*N*-dmba=*N*,*N*′-dimethylbenzylamine; isn: isonicotinamide) against the intracellular amastigote forms of both L. amazonensis and T. cruzi (47), which were used to synthesize the CP2 derivative evaluated here.

The metal-based compound CP2 has shown leishmanicidal activity against L. amazonensis promastigotes and intracellular amastigotes. Although the observed leishmanicidal effect of CP2 (IC_50_ = 10.1 μM, SI = 49.9) toward intracellular amastigotes was half of that obtained with amphotericin B (IC_50_ = 4.9 μM, SI = 4.7), the cyclopalladated compound displayed 10-fold less toxicity to the peritoneal macrophages, as denoted by the SI values; additionally, CP2 was able to reduce the number of amastigotes in infected macrophages by 68.5%, similarly to amphotericin B in the *in vitro* assays carried out (Fig. 2A). Regarding the spectrum of activity, CP2 (IC_50_ = 2.3 μM; SI = 225) was 2-fold more effective than benznidazol (IC_50_ = 5.3 μM; SI = 187.2) against amastigotes of T. cruzi, making this compound very attractive for further evaluation in *in vivo* studies for its potential application in the therapy of Chagas disease.

The high potency of CP2 (IC_50_ = 10 μM) against intracellular amastigotes of L. amazonensis is in accordance with the Drugs for Neglected Diseases Initiative guidelines (48); this compelled us to explore its efficacy in an *in vivo* cutaneous leishmaniasis model. Treatment of L. amazonensis-infected mice with 0.35 mg/kg/day of CP2 through intraperitoneal administration, the first commonly used route in evaluating the potential effect of a compound, led to a significant reduction in parasite load in foot lesions (80%, as determined from the LDU). Although the reduction in the parasite burden in L. amazonensis-infected mice treated with CP2 was similar to that observed with amphotericin B (83%), the concentration of the amphotericin B was five times higher than that of CP2 and caused severely toxic effects to the animals, including weight loss at the beginning of treatment, cachexia, and nosebleeds, which were not observed in the group treated with CP2. Thus, at day 11 we followed the amphotericin B manufacturer's instruction of reducing the amphotericin B dosage in cases of severe toxic effects by switching from a daily to an alternate-day regimen until the end of the therapy course. Although CP2 did not result in a total cure in infected mice, it is worth noting that the BALB/c strain is highly susceptible to L. amazonensis infection (49), so it would be harder to eradicate. The plasma levels of hepatic and renal biomarkers after the treatment with CP2 did not show hepatobiliary disorders or renal failure (Fig. 5), which corroborates the low toxicity of cyclopalladated compounds reported in the literature (41). The altered dose regimen for amphotericin B might also have influenced the absence of toxic effects since hepatic and renal toxicity have been previously reported (50, 51). It is worth mentioning that *in silico* analysis to determine the safety and possible oral effectiveness of CP2 was carried out using the admetSAR tool (52) (Table 2). A compound is classified as orally effective when it demonstrates good absorption. Thus, the evaluated ADMET properties predicted that CP2 presents 92.96% of permeability for human intestinal absorption and 87.19% of permeability for the blood-brain barrier. With regard to metabolism, this molecule was classified as a nonsubstrate and a noninhibitor of a series of cytochrome P450 isoforms, although CP2 was shown to be a substrate for CYP450 3A4 (57.82%). Toxicity was also analyzed, and CP2 demonstrated the absence of carcinogenic toxicity and cardiotoxicity (noninhibitor for hERG). CP2 is also predicted as a mere class III risk for acute toxicity since it showed value for oral rat acute toxicity (LD_50_) of 2.7317 mg/kg (compounds with an LD_50_ greater than 500 mg/kg) (53). Taken together, these data suggest that CP2 is safe and orally absorbed.

Some mechanistic studies have demonstrated the ability of different metal compounds containing platinum, cadmium, copper, and gold to inhibit the activity of type 1 or 2 DNA topoisomerases (19–22). This could be due to the ability of inorganic drugs to coordinate the active site of the enzyme, blocking the enzyme-DNA interaction. Alternatively, the drugs could be coordinated to residues close to the active site, resulting in an altered enzyme structure (16); indeed, the antileishmanial pentavalent antimonial sodium stibogluconate inhibits the *Ld*Topo1B (54). Based on this information, we decided to investigate whether CP2 is able to inhibit the activity of *Ld*Topo1B DNA, the only one currently available for this kind of analysis (55).

Our *in vitro* results have shown that the relaxation activity of *Ld*Topo1B is inhibited by CP2 and that the inhibition is dose dependent (Fig. 6 and 7). This suggests that this enzyme is targeted through the mechanism of CP2 treatment against Leishmania parasites, which has yet to be precisely elucidated. Preincubation of CP2 with the *Ld*topo1B enzyme increased the inhibitory ability of the relaxation reaction, whereas incubation with DNA did not affect relaxation, demonstrating that CP2 has a high affinity for *Ld*topo1B (Fig. 7). Together, these data suggest that CP2 can be classified as a catalytic DNA topoisomerase inhibitor (25).

It is well known that inhibition of DNA topoisomerases should affect mainly cell cycling cells such cancer cells (22, 56–59), but the toxicity assays performed here against the LM3 cells demonstrated that CP2 presents low selectivity for these cancer cells compared to Leishmania amastigotes, which could be indicative of the selectivity to the parasite's molecular target. It is worth noting that structural differences between human DNA topoisomerase I and *Ld*Topo1B might be the reason of the higher selectivity of CP2 to the parasite over the LM3 cells. The human Topo1 is a monomeric enzyme composed of 765 residues, whereas the *Ld*Topo1B is expressed from two open reading frames to produce a heterodimer consisting of a large 635-residue subunit (*Ld*TOP1L) and a small 262-residue subunit (*Ld*TOP1S) (30). In addition, CP2 was 2-fold less cytotoxic (71 μM) to LM3 than the antitumor drug cisplatin (30.3 μM), a potential inhibitor of human DNA topoisomerase II (60). This suggests that neither of the two human DNA topoisomerases might be a potent CP2 target. Altogether, the data presented here might explain the absence of toxicity reported in our *in vivo* assay.

Our data indicate that CP2 is very efficient due to its excellent biological activity against L. amazonensis, as well as T. cruzi, an indication of its potential wide action spectrum, high selectivity index, and great capacity to decrease parasite burden in *in vivo* experiments. In addition, its specific inhibitory effect on the topoisomerization of DNA catalyzed by *Ld*Topo1B makes CP2 a good candidate for further exploration as a potential drug candidate against leishmaniasis and Chagas disease.

## MATERIALS AND METHODS

### Compounds.

Cyclometallated species represent an important class of compounds in organometallic chemistry due to their properties allowing many applications, including their use for therapeutic purposes such as antiprotozoal and anticancer treatments (61). The synthesis of CP2 and its precursors was carried out at room temperature. The binuclear cyclopalladated complex [Pd(dmba)(μ-N_3_)]_2_, here denominated CP2, was obtained as previously described (61). In summary, CP2 was prepared starting from 1.25 mmol of the compound [Pd(dmba)(μ-Cl)]_2_ (62, 63) and 2.5 mmol of NaN_3_ (Riedel-de Haën) in an acetone solution (Mallinckrodt). The mixture was stirred for 1 h, and the yellow solid was filtered off, washed with water and pentane (Merck), and dried *in vacuo*. The yield was 87%, with a melting point of 187°C (decomposition). The resulting CP2 and the reference drugs pentamidine isethionate (Sigma-Aldrich), amphotericin B (Cristalia, São Paulo, Brazil), and benznidazole (Sigma-Aldrich) were dissolved in DMSO (Sigma-Aldrich) and further diluted in culture media. Stock solutions were kept at −20°C. For *in vivo* experiments, stock solutions were prepared in PBS solution as a vehicle. The drugs were prepared daily, immediately before use.

### Biological assays for parasites.

Promastigotes of L. amazonensis strain MPRO/BR/1972/M1841-LV-79 and epimastigotes of T. cruzi strain Y were maintained at 28°C in a liver-infusion tryptose (LIT) medium supplemented with 10% heat-inactivated fetal bovine serum (FBS; Gibco/Invitrogen) (64).

### Evaluation of *in vitro* antiprotozoal activity.

To determine the IC_50_ (the half-maximal inhibitory concentration), we used the 3-(4,5-dimethylthiazol-yl)-2,5-diphenyltetrazolium bromide (MTT; Sigma-Aldrich) colorimetric assay for L. amazonensis promastigotes and T. cruzi epimastigotes forms as previously described (47). Cells were plated in 96-well plates (TPP, Trasadingen, Switzerland) at a density of 10^7^ parasites/ml (in a final volume of 100 μl) and incubated at 28°C in the presence of increasing concentrations of CP2 or reference drugs (from 0.5 to 100 μM) for 72 h for L. amazonensis and T. cruzi. The absorbance was then read in a plate reader (Robonik, Maharashtra, India) at 490 nm for Leishmania species according to the protocol as previously established in our laboratory and at 595 nm for T. cruzi (4, 47). The assays were carried out in triplicates, and data analysis and calculations of their IC_50_s were performed using the software Origin 7.0 (65).

### Evaluation of cytotoxicity on murine macrophages and murine LM3 tumor cell lines.

The cytotoxicity toward murine macrophages or LM3 cells was determined as previously described (66). In summary, for mouse peritoneal macrophages, the cells were collected from adult male Swiss albino mice (20 to 35 g) and seeded in 96-well flat-bottom plates at a density of 10^5^ cells/well (100 μl/well) in RPMI 1640 medium supplemented with 10% heat-inactivated FBS, 25 mM HEPES, and 2 mM l-glutamine, followed by incubation for 24 h at 37°C in a 5% CO_2_-air mixture. For *in vitro* assay against murine mammary LM3, cells were maintained in minimum essential medium (Sigma) supplemented with 10% heat-inactivated FBS, 2 mM l-glutamine, and gentamicin at 50 µg/ml in plastic flasks (TPP) at 37°C in a 5% CO_2_-air mixture. The number of cells was determined by the trypan blue dye exclusion method. LM3 cells were adjusted to a density of 5 × 10^4^ cells/ml and transferred to each well of a 96-well flat-bottom plate. All cells used for cytotoxicity studies were preincubated for 24 h to allow the cells to adapt prior to the addition of the test compounds. Then, the supernatants were removed, and new medium was added containing different concentrations of CP2 or the reference drugs ranging from 1.25 to 100 μM.

Positive (with amphotericin B, pentamidine isethionate, benznidazole, or cisplatin) and negative (without drugs) controls were included. Plates were then incubated under the same conditions for 24 h to be used for a MTT colorimetric assay, which was carried out as previously described (67). The absorbance was then read in a 96-well plate reader at 595 nm. The drug concentration that corresponds to 50% of cell growth inhibition is expressed as the 50% cytotoxic concentration (CC_50_). The cytotoxicity for host cells and protozoan species were compared and expressed as the selectivity index (SI), which was defined as the ratio of the CC_50_ for macrophages to the IC_50_ for protozoan species.

### Differentiation of T. cruzi epimastigote and trypomastigote forms.

*In vitro* differentiation of T. cruzi was performed according to the literature (68). Briefly, epimastigotes from a stationary culture in LIT medium were harvested by centrifugation at 10,000 × *g* for 15 min at 10°C (Eppendorf) and cultured in freshly prepared LIT medium for another 48 h at 28°C supplemented with triatomine artificial urine (TAU; prepared in 190 mM NaCl, 8 mM phosphate buffer (pH 6.0), 17 mM KCl, 2 mM CaCl_2_, 2 mM MgCl_2_, and 0.6 mM NaHCO_3_). Cultures were then incubated in a medium supplemented with TAU and 10 mM l-proline (TAUP medium) at 27°C for 10 days.

### Evaluation of *in vitro* activity against L. amazonensis and T. cruzi intracellular amastigotes.

To determine the leishmanicidal and trypanocidal activity of CP2 and reference drugs against intracellular amastigotes of L. amazonensis and T. cruzi, murine peritoneal macrophages were plated at a density of 3 × 10^5^ cells/well on coverslips (13 mm in diameter), previously arranged in a 24-well plate in RPMI 1640 medium supplemented with 10% heat-inactivated FBS. The cells were then allowed to adhere for 4 h at 37°C in 5% CO_2_ (69). Adherent macrophages were infected with L. amazonensis promastigotes or T. cruzi trypomastigotes in the stationary growth phase (using ratios of 5:1 and 10:1 parasites per macrophage, respectively) at 37°C in 5% CO_2_ for 4 h for L. amazonensis and 24 h for T. cruzi. The noninternalized parasites were then removed by washing, and infected cultures were incubated in RPMI 1640 medium for 24 h at 37 ± 2°C in 5% CO_2_ to allow parasite multiplication. The infected cells were then treated with different concentrations of CP2, pentamidine isethionate, amphotericin B, or benznidazole for 24 h. After incubation, the cells were fixed with methanol, stained with Giemsa, and examined using optical microscopy (Opton). The infection index was determined by multiplying the percentage of infected macrophages by the mean number of amastigotes per infected cells. The concentration that resulted in a 50% decrease of growth inhibition compared to the control was determined by regression analysis and expressed as IC_50_ in μM (47).

### Antileishmanial *in vivo* assays.

For evaluation of *in vivo* leishmanicidal activity of CP2, female BALB/c mice (20 to 25 g; 4 weeks old; CEMIB, UNICAMP) were subcutaneously inoculated at the right hind-footpad with 10^7^ infective promastigotes of L. amazonensis in 10 μl of PBS (early stationary phase of growth). After 8 weeks, when the lesions presented a diameter of 5 to 7 mm, the animals were randomly separated into four groups of eight animals each. Treated animals received 0.20 or 0.35 mg/kg (body weight)/day of CP2 for 35 days. Stock solutions of CP2 were prepared daily in 1× PBS after solubilization in DMSO (final concentration, 0.1%). Negative controls correspond to infected and nontreated mice, as well as those that received the same number of injections with 1× PBS (vehicle). As positive control, a group of mice received 2 mg/kg/day of amphotericin B, and a group of uninfected and untreated mice (healthy animals) was used for the analysis. The compound was administered intraperitoneally daily for 35 days. Infection was monitored three times a week by measuring the thickness of the foot lesions with a dial caliper (Mitutoyo Corp., Japan). Treatment efficacy was determined by measuring the parasite burden of infected feet using the LDU index (70).

### LDU index.

BALB/c mice were euthanized by CO_2_ asphyxiation, and necropsies were performed for parasitological diagnoses of tissue smears. Parasites were counted from Giemsa-stained tissue impression smears and examined using optical microscopy for the identification of amastigote forms of Leishmania. The parasite density evaluation was performed, and the results were expressed as the LDU index, according to Stauber (70) and other authors (71–73); these data correspond to the number of amastigotes in 1,000 nucleated cells/organ weight.

### *In silico* prediction of CP2 pharmacokinetic and toxicity properties.

For determination of the safety and potential of oral absorption of CP2, the ADMET properties were evaluated by using the admetSAR tool (52). The SMILES (simplified molecular-input line-entry system) used for *in silico* analysis was as follows: [N][N]N1[Pd]23([N](C([H])([H])[H])(C([H])([H])[H])C([H])([H])C4C3C(C(C(C4[H])[H])[H])[H])[Pd]15([N](C([H])([H])[H])(C([H])([H])[H])C([H])([H])C6C5C(C(C(C6[H])[H])[H])[H])N2[N][N].

### Toxicity for mice assays.

Plasma concentrations of total bilirubin, ALT and AST, ALP, urea, and creatinine were determined in BALB/c mice at the end of the treatments using commercial kits (Labtest Diagnostica S.A., Brazil). The following principles were used in these methods to determine concentration: AST, continuous kinetic monitoring, coupled with malate dehydrogenase (74); ALT, continuous kinetic monitoring, coupled with lactate dehydrogenase (75); ALP, colorimetric method based on the rate of hydrolysis of p-nitrophenyl phosphate (76); total bilirubin, azobilirubin formation in the presence of diazotized dichloroaniline (77, 78); creatinine, chromogen formation with picrate in alkaline medium (79); and urea, the urease method (80). Blood plasma samples, collected from blood samples obtained by cardiac puncture in the presence of heparin at 5,000 IU/ml (Hemofol; Cristalia) and centrifuged at 2,500 × *g* for 10 min at 4°C, were stored at −20°C until ready for analyzing the changes in the biomarkers of hepatic and renal function. Assays were performed by spectrophotometric system identification in a semiautomated biochemical analyzer.

### *Ld*topo1B expression.

To express the full-length L. donovani topoisomerase 1B (*Ld*topo1B), the plasmid pESC*LdTOP1A-LdTOP1B-URA* (kindly donated by Rafael Balaña Fouce), with the inducible promoters GAL1 and GAL10 was used. This plasmid was used to transform a Saccharomyces cerevisiae EKY3 strain deficient in DNA topoisomerase I activity (*MAT*α *ura3-52 his3Δ200 leu2Δ1 trp163 top1 D*::TRP1), as previously described (56). Transformed cells were selected on solid synthetic complement (SC)-uracil medium plus 2% dextrose at 30°C, transferred into liquid SC-uracil medium plus 2% dextrose, and grown overnight at 30°C and 140 rpm. At an optical density at 600 nm between 1 and 3, the cells were diluted 1:100 in SC-uracil plus 2% raffinose and induced for protein production by the application of 2% galactose for 6 h. Cells were harvested by centrifugation (4,000 × *g* for 10 min at 4°C), washed with cold water, resuspended at the ratio of 1 g (wet weight) of cells/2 ml of TEEG buffer (50 mM Tris-HCl [pH 7.4], 1 mM EDTA, 1 mM EGTA, 10% [vol/vol] glycerol), and supplemented with a protease inhibitor cocktail (sodium bisulfite, 0.1 mg/ml; NaF, 0.8 mg/ml; 2× Complete Mini) according to the manufacturer's instructions (Roche Molecular Biochemicals, catalog no. 1836153). The cells were stored at −80°C.

### *Ld*topo1B protein purification.

After the cells were thawed, 0.5 volumes of 425- to 600-μm glass beads were added to the cell suspension, and the cells were disrupted with 30 repetitions of 30-s vortexing each time, followed by a 30-s incubation on ice. The lysate was clarified by centrifugation (15,000 × *g*, 30 min), and the proteins were subjected to successive ammonium sulfate fractionations. A 35% saturation was obtained by adding ammonium sulfate to the extract (19.4 g/100 ml) and permitting the mixture to dissolve by rocking at 4°C for 60 min. The precipitates were removed by centrifugation (15,000 × *g*, 30 min), and the supernatant was then adjusted to 75% saturation in solid ammonium sulfate (25.4 g/100 ml) with gentle rocking at 4°C overnight. The precipitates were recovered by centrifugation (15,000 × *g*, 30 min) and resuspended in TEEG buffer–200 mM KCl, loaded onto a P11 resin equilibrated in the same buffer. Elution of *Ld*topo1B was performed at 4°C in TEEG buffer supplemented with protease inhibitor cocktail, 0.1 mg/ml sodium bisulfite, and 0.8 mg/ml sodium fluoride, with a discontinuous gradient of KCl (0.2, 0.4, 0.6, 0.8, and 1 M). *Ld*topo1B was eluted at between 0.4 and 0.6 M KCl.

### *Ld*topo1B relaxation assay.

In order to determine the concentration of the eluted *Ld*topo1B (in U/μl), 1 μl of enzyme solution was diluted 3-, 9-, 27-, and 81-fold in reaction buffer (20 mM Tris–HCl, 0.1 mM Na_2_EDTA, 10 mM CaCl_2_, 50 µg/ml acetylated bovine serum albumin [BSA], and 150 mM KCl [pH 7.5]) before the addition of 0.5 µg of negatively supercoiled pBluescript KSII(+) DNA (standard conditions) in a 30-μl reaction volume. Each reaction of *Ld*topo1B was incubated at 37°C for 30 min. One unit of enzyme is defined as the amount required to completely relax 0.5 μg of negative supercoiled plasmid DNA in 30 min at 37°C.

One unit of the eluted *Ld*topo1B was assayed in a 20-μl reaction volume containing negatively supercoiled pBluescript KSII(+) DNA according to the standard conditions described above. Increasing concentrations of CP2 (0 to 300 µM) were added to each reaction mixture, which were stopped with a final concentration of 0.5% sodium dodecyl sulfate (SDS) after 30 min or after each time course point (1, 7.5, 15, and 30 min) at 37°C. The samples were electrophoresed in a horizontal 1% agarose gel in 50 mM Tris, 45 mM boric acid, and 1 mM EDTA, which was then stained with ethidium bromide (5 µg/ml), destained with water, and photographed under UV illumination. Assays were performed at least three times on one representative gel.

### *Ld*topo1B cleavage assays.

The oligonucleotide CL1 (5′-GAAAAAAGACTTAG-3′) radiolabeled with [γ-^32^P]ATP at its 5′ end was annealed with a 2-fold molar excess of CL2 complementary strand (5′-TAAAAATTTTTCTAAGTCTTTTTTC-3′) (20) to produce a partially duplex substrate with a single-strand extension of 11 nucleotides called “suicide substrate” (Fig. 7). The cleavage reactions were carried out by incubating a 20 nM concentration of the suicide substrate with 2.5 U of enzyme in 20 mM Tris-HCl (pH 7.5), 0.1 mM Na_2_EDTA, 10 mM CaCl_2_, 50 μg/ml acetylated BSA, and 150 mM KCl at 23°C. A 5-μl sample of the reaction mixture was taken before addition of the enzyme. At various time points, 5-μl aliquots were taken, and the reaction stopped in each one by using 0.5% SDS. After ethanol precipitation, the samples were resuspended in 5 μl of trypsin at 1 mg/ml, followed by incubation at 37°C for 30 min. Reaction products were analyzed in denaturing 20% acrylamide–7 M urea gels and visualized with a PhosphorImager.

### Ethics statement.

Animal experiments were approved by the Ethics Committee for Animal Experimentation of São Paulo State University (UNESP), the School of Pharmaceutical Sciences (CEUA/FCF/CAr, 20/2013 and 37/2013; CEUA/FCF/CAr, 25/2014 and 30/2014) in agreement with the guidelines of the Sociedade Brasileira de Ciência de Animais de Laboratorio (SBCAL) and of the Conselho Nacional de Controle da Experimentação Animal (CONCEA).

### Statistical analysis.

The statistical differences between groups were evaluated using one-way analysis of variance, followed by the Student-Newman-Keuls multiple -comparison test (using GraphPad InStat software). Differences were considered significant when *P* values were ≤0.05.

## References

[1] Murray HW, Berman JD, Davies CR, Saravia NG 2005 Advances in leishmaniasis. Lancet 366:1561–1577. doi:10.1016/S0140-6736(05)67629-5. 16257344

[2] Singh N, Kumar M, Singh RK 2012 Leishmaniasis: current status of available drugs and new potential drug targets. Asian Pac J Trop Med 5:485–497. doi:10.1016/S1995-7645(12)60084-4. 22575984

[3] Torres FA, Passalacqua TG, Velásquez AM, de Souza RA, Colepicolo P, Graminha MA 2014 New drugs with antiprotozoal activity from marine algae: a review. Braz J Pharmacogn 24:265–276. doi:10.1016/j.bjp.2014.07.001.

[4] Santos VA, Regasini LO, Nogueira CR, Passerini GD, Martinez I, Bolzani VS, Graminha MA, Cicarelli RM, Furlan M 2012 Antiprotozoal sesquiterpene pyridine alkaloids from *Maytenus ilicifolia*. J Nat Prod 75:991–995. doi:10.1021/np300077r. 22559947

[5] Meheus F, Balasegaram M, Olliaro P, Sundar S, Rijal S, Faiz MA, Boelaert M 2010 Cost-effectiveness analysis of combination therapies for visceral leishmaniasis in the Indian subcontinent. PLoS Negl Trop Dis 4:e818. doi:10.1371/journal.pntd.0000818. 20838649 PMC2935395

[6] Van Griensven J, Diro E 2012 Visceral leishmaniasis. Infect Dis Clin North Am 26:309–322. doi:10.1016/j.idc.2012.03.005. 22632641

[7] Sundar S, Rai M 2002 Advances in the treatment of leishmaniasis. Curr Opin Infect Dis 15:593–598. doi:10.1097/00001432-200212000-00007. 12821836

[8] Singh S, Sivakumar R 2004 Challenges and new discoveries in the treatment of leishmaniasis. J Infect Chemother 10:307–315. doi:10.1007/s10156-004-0348-9. 15614453

[9] Dorlo TPC, Balasegaram M, Beijnen JH, de Vries PJ 2012 Miltefosine: a review of its pharmacology and therapeutic efficacy in the treatment of leishmaniasis. J Antimicrob Chemother 67:2576–2597. doi:10.1093/jac/dks275. 22833634

[10] World Health Organization. 2010 Control of the leishmaniases: report of a meeting of the WHO Expert Committee on the Control of Leishmaniasis. World Health Organization, Geneva, Switzerland.

[11] Barrett MP, Croft SL 2012 Management of trypanosomiasis and leishmaniasis. Br Med Bull 104:175–196. doi:10.1093/bmb/lds031. 23137768 PMC3530408

[12] Farrell NP, Williamson J, McLaren DJM 1984 Trypanocidal and antitumour activity of platinum-metal and platinum-metal-drug dual-function complexes. Biochem Pharmacol 33:961–971. doi:10.1016/0006-2952(84)90501-X. 6538791

[13] Zinsstag J, Brun R, Craciunescu DG, Parrondo Iglesias E 1991 In vitro activity of organometallic complexes of iridium, platinum, and rhodium on *Trypanosoma b. gambiense*, *T. b. rhodesiense*, and *T. b. brucei*. Trop Med Parasitol 42:41–44. 2052855

[14] Sánchez-Delgado RA, Anzellotti A 2004 Metal complexes as chemotherapeutic agents against tropical diseases: trypanosomiasis, malaria, and leishmaniasis. Mini-Reviews Med Chem 4:23–30. doi:10.2174/1389557043487493. 14754440

[15] Fricker SP, Mosi RM, Cameron BR, Baird I, Zhu Y, Anastassov V, Cox J, Doyle PS, Hansell E, Lau G, Langille J, Olsen M, Qin L, Skerlj R, Wong RSY, Santucci Z, McKerrow JH 2008 Metal compounds for the treatment of parasitic diseases. J Inorg Biochem 102:1839–1845. doi:10.1016/j.jinorgbio.2008.05.010. 18684510

[16] Navarro M, Gabbiani C, Messori L, Gambino D 2010 Metal-based drugs for malaria, trypanosomiasis, and leishmaniasis: recent achievements and perspectives. Drug Discov Today 15:1070–1078. doi:10.1016/j.drudis.2010.10.005. 20974285

[17] Matsuo AL, Silva LS, Torrecilhas AC, Pascoalino BS, Ramos TC, Rodrigues EG, Schenkman S, Caires AC, Travassos LR 2010 In vitro and in vivo trypanocidal effects of the cyclopalladated compound 7a, a drug candidate for treatment of Chagas' disease. Antimicrob Agents Chemother 54:3318–3325. doi:10.1128/AAC.00323-10. 20479201 PMC2916297

[18] Paladi CD, Pimentel IA, Katz S, Cunha RL, Judice WA, Caires AC, Barbiéri CL 2012 In vitro and in vivo activity of a palladacycle complex on *Leishmania amazonensis*. PLoS Negl Trop Dis 6:e1626. doi:10.1371/journal.pntd.0001626. 22616018 PMC3352823

[19] Casini A, Kelter G, Gabbiani C, Cinellu MA, Minghetti G, Fregona D, Fiebig HH, Messori L 2009 Chemistry, antiproliferative properties, tumor selectivity, and molecular mechanisms of novel gold(III) compounds for cancer treatment: a systematic study. J Biol Inorg Chem 14:1139–1149. doi:10.1007/s00775-009-0558-9. 19543922

[20] Castelli S, Vassallo O, Katkar P, Che CM, Sun RW, Desideri A 2011 Inhibition of human DNA topoisomerase IB by a cyclometalated gold III compound: analysis on the different steps of the enzyme catalytic cycle. Arch Biochem Biophys 516:108–112. doi:10.1016/j.abb.2011.10.008. 22033340

[21] Wu X, Yalowich JC, Hasinoff BB 2011 Cadmium is a catalytic inhibitor of DNA topoisomerase II. J Inorg Biochem 105:833–838. doi:10.1016/j.jinorgbio.2011.02.007. 21497582 PMC3091975

[22] Neves AP, Pereira MX, Peterson EJ, Kipping R, Vargas MD, Silva FP Jr, Carneiro JW, Farrell NP 2013 Exploring the DNA binding/cleavage, cellular accumulation and topoisomerase inhibition of 2-hydroxy-3-(aminomethyl)-1,4-naphthoquinone mannich bases and their platinum(II) complexes. J Inorg Biochem 119:54–64. doi:10.1016/j.jinorgbio.2012.10.007. 23186648

[23] Brata Das B, Sen N, Ganguly A, Majumder HK 2004 Reconstitution and functional characterization of the unusual bi-subunit type I DNA topoisomerase from *Leishmania donovani*. FEBS Lett 565:81–88. doi:10.1016/j.febslet.2004.03.078. 15135057

[24] Chawla B, Madhubala R 2010 Drug targets in leishmania. J Parasit Dis 34:1–13. doi:10.1007/s12639-010-0006-3. 21526026 PMC3081701

[25] D'Annessa I, Castelli S, Desideri A 2015 Topoisomerase 1B as a target against leishmaniasis. Mini Rev Med Chem 15:203–210. doi:10.2174/138955751503150312120912. 25769969

[26] Chakraborty AK, Gupta A, Majumder HK 1993 A type 1 DNA topoisomerase from the kinetoplast hemoflagellate *Leishmania donovani*. Indian J Biochem Biophys 30:257–263. 8144168

[27] Riou GF, Gabillot M, Douc-Rasy S, Kayser A, Barrois M 1983 A type I DNA topoisomerase from *Trypanosoma cruzi*. Eur J Biochem 134:479–484. doi:10.1111/j.1432-1033.1983.tb07592.x. 6309514

[28] Bodley AL, Chakraborty AK, Xie S, Burri C, Shapiro TA 2003 An unusual type IB topoisomerase from African trypanosomes. Proc Natl Acad Sci U S A 100:7539–7544. 12810956 10.1073/pnas.1330762100 PMC164622

[29] Broccoli S, Marquis JF, Papadopoulou B, Olivier M, Drolet M 1999 Characterization of a *Leishmania donovani* gene encoding a protein that closely resembles a type IB topoisomerase. Nucleic Acids Res 27:2745–2752. doi:10.1093/nar/27.13.2745. 10373592 PMC148484

[30] Davies DR, Mushtaq A, Interthal H, Champoux JJ, Hol WGJ 2006 The structure of the transition state of the heterodimeric topoisomerase I of *Leishmania donovani* as a vanadate complex with nicked DNA. J Mol Biol 357:1202–1210. doi:10.1016/j.jmb.2006.01.022. 16487540

[31] Rosenberg B, Vancamp L, Krigas T 1965 Inhibition of cell division in *Escherichia coli* by electrolysis products from a platinum electrode. Nature 205:698–699. doi:10.1038/205698a0. 14287410

[32] Otero L, Vieites M, Boiani L, Denicola A, Rigol C, Opazo L, Olea-Azar C, Maya JD, Morello A, Krauth-Siegel RL, Piro OE, Castellano E, González M, Gambino D, Cerecetto H 2006 Novel antitrypanosomal agents based on palladium nitrofurylthiosemicarbazone complexes: DNA and redox metabolism as potential therapeutic targets. J Med Chem 49:3322–3331. doi:10.1021/jm0512241. 16722651

[33] Vieites M, Otero L, Santos D, Toloza J, Figueroa R, Norambuena E, Olea-Azar C, Aguirre G, Cerecetto H, González M, Morello A, Maya JD, Garat B, Gambino D 2008 Platinum(II) metal complexes as potential anti-*Trypanosoma cruzi* agents. J Inorg Biochem 102:1033–1043. doi:10.1016/j.jinorgbio.2007.12.005. 18226837

[34] Caires AC, Almeida ET, Mauro AE, Hemerly JP, Valentini SR 1999 Síntese e atividade citotóxica de alguns azido-ciclopaladados estabilizados com ligantes bifosfínicos. Quim Nova 22:329–334. doi:10.1590/S0100-40421999000300008.

[35] Santos D, Parajón-Costa B, Rossi M, Caruso F, Benítez D, Varela J, Cerecetto H, González M, Gómez N, Caputto ME, Moglioni AG, Moltrasio GY, Finkielsztein LM, Gambino D 2012 Activity on *Trypanosoma cruzi*, erythrocytes lysis and biologically relevant physicochemical properties of Pd(II) and Pt(II) complexes of thiosemicarbazones derived from 1-indanones. J Inorg Biochem 117:270–276. doi:10.1016/j.jinorgbio.2012.08.024. 23063173

[36] Vieites M, Smircich P, Guggeri L, Marchán E, Gómez-barrio A, Navarro M, Garat B, Gambino D 2009 Synthesis and characterization of a pyridine-2-thiol N-oxide gold(I) complex with potent antiproliferative effect against *Trypanosoma cruzi* and *Leishmania* sp. insight into its mechanism of action. Q J Inorg Biochem 103:1300–1306. doi:10.1016/j.jinorgbio.2009.02.011. 19361864

[37] Croft SL, Neal RA, Craciunescu DG, Certadfombona G 1992 The activity of platinum, iridium, and rhodium drug complexes against *Leishmania donovani*. Trop Med Parasitol 43:24–28. 1598504

[38] Rodriguez-Cabezas MN, Mesa-Valle CM, Azzouz S, Moraleda-Lindez V, Craciunescu D, Gutierrez-Rios MT, De Frutos MI, Osuna A 2001 In vitro and in vivo activity of new rhodium(III) complexes against *Leishmania donovani*. Pharmacology 63:112–119. doi:10.1159/000056121. 11490204

[39] Silva ML, Neto AF, Cardoso SA, Albuquerque S, Miller J 2002 Synthesis and “in vitro” trypanocidal activity evaluation of some organo-iron compounds. Met drugs 8:329–332. doi:10.1155/MBD.2002.329. PMC2365288 18476014

[40] Velásquez AM, Francisco AI, Kohatsu AA, Silva FA, Rodrigues DF, Teixeira RG, Chiari BG, De Almeida MG, Isaac VL, Vargas MD, Cicarelli RM 2014 Synthesis and tripanocidal activity of ferrocenyl and benzyl diamines against *Trypanosoma brucei* and *Trypanosoma cruzi*. Bioorganic Med Chem Lett 24:1707–1710. doi:10.1016/j.bmcl.2014.02.046. 24630563

[41] Rodrigues EG, Silva LS, Fausto DM, Hayashi MS, Dreher S, Santos EL, Pesquero JB, Travassos LR, Caires ACF 2003 Cyclopalladated compounds as chemotherapeutic agents: antitumor activity against a murine melanoma cell line. Int J Cancer 107:498–504. doi:10.1002/ijc.11434. 14506753

[42] de Souza RA, Stevanato A, Treu-Filho O, Netto AV, Mauro AE, Castellano EE, Carlos IZ, Pavan FR, Leite CQ 2010 Antimycobacterial and antitumor activities of palladium(II) complexes containing isonicotinamide (isn): X-ray structure of trans-[Pd(N3)2(isn)2]. Eur J Med Chem 45:4863–4868. doi:10.1016/j.ejmech.2010.07.057. 20724041

[43] Moro AC, Mauro AE, Netto AV, Ananias SR, Quilles MB, Carlos IZ, Pavan FR, Leite CQ, Hörner M 2009 Antitumor and antimycobacterial activities of cyclopalladated complexes: X-ray structure of [Pd(C2,N-dmba)(Br)(tu)] (dmba=N,N-dimethylbenzylamine, tu=thiourea). Eur J Med Chem 44:4611–4615. doi:10.1016/j.ejmech.2009.06.032. 19632008

[44] Moro AC, Urbaczek AC, De Almeida ET, Pavan FR, Leite CQ, Netto AV, Mauro AE 2012 Binuclear cyclopalladated compounds with antitubercular activity: synthesis and characterization of [{Pd(C2,N-dmba)(X)} 2(μ-bpp)] (X=Cl, Br, NCO, N 3; bpp=1,3-bis(4-pyridyl)propane). J Coord Chem 65:1434–1442. doi:10.1080/00958972.2012.673718.

[45] Da Rocha MC, Santana AM, Ananias SR, De Almeida ET, Mauro AE, Placeres MC, Carlos IZ 2007 Cytotoxicity and immune response induced by organopalladium(II) compounds in mice bearing Ehrlich ascites tumour. J Braz Chem Soc 18:1473–1480. doi:10.1590/S0103-50532007000800004.

[46] Spencer J, Rathnam RP, Motukuri M, Kotha AK, Richardson SC, Hazrati A, Hartley JA, Male L, Hursthouse MB 2009 Synthesis of a 1,4-benzodiazepine containing palladacycle with *in vitro* anticancer and cathepsin B activity. Dalt Trans 22:4299–4303. doi:10.1039/b819061e. 19662306

[47] Velásquez AM, De Souza RA, Passalacqua TG, Ribeiro AR, Scontri M, Chin CM, De Almeida L, Del Cistia ML, Da Rosa JA, Mauro AE, Graminha MA 2016 Antiprotozoal activity of the cyclopalladated complexes against *Leishmania amazonensis* and *Trypanosoma cruzi*. J Braz Chem Soc 27:1032–1039.

[48] Katsuno K, Burrows JN, Duncan K, van Huijsduijnen RH, Kaneko T, Kita K, Mowbray CE, Schmatz D, Warner P, Slingsby BT 2015 Hit and lead criteria in drug discovery for infectious diseases of the developing world. Nat Rev Drug Discov 14:751–758. doi:10.1038/nrd4683. 26435527

[49] Lainson R, Shaw JJ 1987 Evolution, classification, and geographical distribution, p 1–120. *In* Killick-Kendrick R, Peters W (ed), The leishmaniases in biology and medicine. Academic Press, London, United Kingdom.

[50] Wortmann G, Zapor M, Ressner R, Fraser S, Hartzell J, Pierson J, Weintrob A, Magill A 2010 Liposomal amphotericin B for treatment of cutaneous leishmaniasis. Am J Trop Med Hyg 83:1028–1033. doi:10.4269/ajtmh.2010.10-0171. 21036832 PMC2963964

[51] Gill J, Sprenger HR, Ralph ED, Sharpe MD 1999 Hepatotoxicity possibly caused by amphotericin B. Ann Pharmacother 33:683–685. doi:10.1345/aph.18181. 10410179

[52] Cheng F, Li W, Zhou Y, Shen J, Wu Z, Liu G, Lee PW, Tang Y 2012 admetSAR: a comprehensive source and free tool for assessment of chemical ADMET properties. J Chem Inform Modeling 52:3099–3105. doi:10.1021/ci300367a. 23092397

[53] Li X, Chen L, Cheng F, Wu Z, Bian H, Xu C, Li W, Liu G, Shen X, Tang Y 2014 In silico prediction of chemical acute oral toxicity using multi-classification methods. J Chem Infect Model 54:1061–1069. doi:10.1021/ci5000467. 24735213

[54] Chakraborty AK, Majumder HK 1988 Mode of action of pentavalent antimonials: specific inhibition of type I DNA topoisomerase of *Leishmania donovani*. Biochem Biophys Res Commun 152:605–611. doi:10.1016/S0006-291X(88)80081-0. 2835038

[55] Balaña-Fouce R, Álvarez-Velilla R, Fernández-Prada C, García-Estrada C, Reguera RM 2014 Trypanosomatids topoisomerase revisited: new structural findings and role in drug discovery. Int J Parasitol Drugs Drug Resist 4:326–337. doi:10.1016/j.ijpddr.2014.07.006. 25516844 PMC4266802

[56] Bjornsti MA, Benedetti P, Viglianti GA, Wang JC 1989 Expression of human DNA topoisomerase I in yeast cells lacking yeast DNA topoisomerase I: restoration of sensitivity of the cells to the antitumor drug camptothecin. Cancer Res 49:6318–6323. 2553253

[57] Kiechle FL, Zhang X 2002 Apoptosis: biochemical aspects and clinical implications. Clin Chim Acta 326:27–45. doi:10.1016/S0009-8981(02)00297-8. 12417095

[58] Walker J, Saravia NG 2004 Inhibition of *Leishmania donovani* promastigote DNA topoisomerase I and human monocyte DNA topoisomerases I and II by antimonial drugs and classical antitopoisomerase agents. J Parasitol 90:1155–1162. doi:10.1645/GE-3347. 15562618

[59] Prada CF, Álvarez-Velilla R, Balaña-Fouce R, Prieto C, Calvo-Álvarez E, Escudero-Martínez JM, Requena JM, Ordóñez C, Desideri A, Pérez-Pertejo Y, Reguera RM 2013 Gimatecan and other camptothecin derivatives poison *Leishmania* DNA-topoisomerase IB leading to a strong leishmanicidal effect. Biochem Pharmacol 85:1433–1440. doi:10.1016/j.bcp.2013.02.024. 23466420

[60] Hasinoff BB, Wu X, Krokhin OV, Ens W, Standing KG, Nitiss JL, Sivaram T, Giorgianni A, Yang S, Jiang Y, Yalowich JC 2005 Biochemical and proteomics approaches to characterize topoisomerase IIα cysteines and DNA as targets responsible for cisplatin-induced inhibition of topoisomerase IIα. Mol Pharmacol 67:937–947. doi:10.1124/mol.104.004416. 15602006

[61] de Almeida ET, Mauro AE, Santana AM, Ananias SR, Netto AV, Ferreira JG, Santos RH 2007 Self-assembly of organometallic Pd(II) complexes via CH3 interactions: the first example of a cyclopalladated compound with herringbone stacking pattern. Inorg Chem Commun (Camb) 10:1394–1398. doi:10.1016/j.inoche.2007.08.020.

[62] Cope AC, Friedrich EC 1968 Electrophilic aromatic substitution reactions by platinum (II) and palladium (II) chlorides on N,N-dimethylbenzylamines. J Am Chem Soc 90:909–913. doi:10.1021/ja01006a012.

[63] Lucca Neto VA, Mauro AE, Caires AC, Ananias SR, de Almeida ET 1999 Synthesis, characterization, and thermal behavior of cyclopalladated compounds of the type [Pd{C6H4CH2N(CH3)2}(μ-X)]2 (X=Cl, NCO, SCN, CN). Polyhedron 18:413–417. doi:10.1016/S0277-5387(98)00310-6.

[64] Silva LH, Nussenzweig V 1953 Sobre uma cepa de *Trypanosoma cruzi* altamente virulenta para o camundongo branco. Folia Clin Biol 20:191–208.

[65] Wass JA 2004 Origin 7.0. Biotech Software Internet Rep 3:130–133. doi:10.1089/152791602321105799.

[66] Passalacqua TG, Dutra LA, De Almeida L, Velásquez AM, Torres Esteves FA, Yamasaki PR, Dos Santos Bastos M, Regasini LO, Michels PA, Da Silva Bolzani V, Graminha MA 2015 Synthesis and evaluation of novel prenylated chalcone derivatives as anti-leishmanial and anti-trypanosomal compounds. Bioorganic Med Chem Lett 25:3342–3345. doi:10.1016/j.bmcl.2015.05.072. 26055530

[67] Dutra LA, De Almeida L, Passalacqua TG, Reis JS, Torres FA, Martinez I, Peccinini RG, Chin CM, Chegaev K, Guglielmo S, Fruttero R, Graminha MA, Dos Santos JL 2014 Leishmanicidal activities of novel synthetic furoxan and benzofuroxan derivatives. Antimicrob Agents Chemother 58:4837–4847. doi:10.1128/AAC.00052-14. 24913171 PMC4136072

[68] Contreras VT, Morel CM, Goldenberg S 1985 Stage specific gene expression precedes morphological changes during *Trypanosoma cruzi* metacyclogenesis. Mol Biochem Parasitol 14:83–96. doi:10.1016/0166-6851(85)90108-2. 3885031

[69] Moore GE, Woods LK 1976 Culture media for human cells: RPMI 1603, RPMI 1634, RPMI 1640, and GEM 1717. Tissue Cult Methods 3:503–508. doi:10.1007/BF00918753.

[70] Stauber LA 1958 Strain of *Leishmania donovani* Host resistance to genus *Leishmania*. Rice Inst Pam 45:80–96.

[71] Seifert K, Juhls C, Salguero FJ, Croft SL 2015 Sequential chemoimmunotherapy of experimental visceral leishmaniasis using a single low dose of liposomal amphotericin B and a novel DNA vaccine candidate. Antimicrob Agents Chemother 59:5819–5823. doi:10.1128/AAC.00273-15. 26055371 PMC4538505

[72] Murray HW, Brooks EB, DeVecchio JL, Heinzel FP 2003 Immunoenhancement combined with amphotericin B as treatment for experimental visceral leishmaniasis. Antimicrob Agents Chemother 47:2513. doi:10.1128/AAC.47.8.2513-2517.2003. 12878513 PMC166064

[73] Mullen AB, Baillie AJ, Carter KC 1998 Visceral leishmaniasis in the BALB/c mouse: a comparison of the efficacy of a nonionic surfactant formulation of sodium stibogluconate with those of three proprietary formulations of amphotericin B. Antimicrob Agents Chemother 42:2722–2725. http://aac.asm.org/content/42/10/2722.long. 9756784 10.1128/aac.42.10.2722 PMC105926

[74] Bergmeyer HU, Bowers GN, Horder M, Moss DW 1977 Provisional recommendation on IFCC methods for the measurement of catalytic concentration of enzymes. 2. IFCC method for aspartate aminotransferase. J Clin Chem Clin Biochem 15:39–51. 954207

[75] Wróblewski F, Ladue JS 1956 Serum glutamic pyruvic transaminase in cardiac and hepatic disease. PSEBM 91:569–571. 10.3181/00379727-91-22330 13323003

[76] Committee on Enzymes of the Scandinavian Society for Clinical Chemistry and Clinical Physiology. 1974 Recommended methods for the determination of four enzymes in blood. Scand J Clin Lab Invest 33:291–306. doi:10.3109/00365517409082499. 4369123

[77] Perry BW, Doumas BT, Bayse DD, Butler T, Cohen A, Fellows W, Garber CC, Howell B, Koch T, Krishnamurthy S, Louderback A, McComb RB, Miller D, Miller RR, Rand RN, Schaffer R 1983 A candidate reference method for determination of bilirubin in serum: test for transferability. Clin Chem 29:297–301. http://www.clinchem.org/content/29/2/297.long. 6821933

[78] Doumas BT, Poon Pat Kwok-Cheung Perry BW 1985 Candidate reference method for determination of total bilirubin in serum: development and validation. Clin Chem 31:1779–1789. http://www.clinchem.org/content/31/11/1779.long. 4053346

[79] Cook JGH 1971 Creatinine assay in the presence of protein. Clin Chim Acta 32:485–486. doi:10.1016/0009-8981(71)90452-9. 5096960

[80] Bernt E, Bergmeyer HU 1965 Urea, p 401–406. *In* Bergmeyer HU (ed), Methods of enzymatic analysis, 2nd ed Academic Press, New York, NY.

